# Rapid Detection of *Fusarium oxysporum* Using Insulated Isothermal PCR and a Rapid, Simple DNA Preparation Protocol

**DOI:** 10.3390/ijms232113253

**Published:** 2022-10-31

**Authors:** Tsai-De Chang, Li-Nian Huang, Yi-Jia Lin, Zhong-Bin Wu, Shang-Han Tsai, Ying-Hong Lin

**Affiliations:** 1 Department of Plant Medicine, National Pingtung University of Science and Technology, Pingtung 91201, Taiwan; 2 Department of Horticulture and Landscape Architecture, National Taitung Jr. College, Taitung 95045, Taiwan; 3 Bachelor Program in Scientific Agriculture, National Pingtung University of Science and Technology, Pingtung 91201, Taiwan

**Keywords:** Fusarium wilt, agricultural management, field diagnosis, reproducibility evaluation, detection performance metrics

## Abstract

We developed an insulated isothermal PCR (iiPCR) method for the efficient and rapid detection of *Fusarium oxysporum* (Fo), which is a fungus that infects various hosts and causes severe crop losses. The Fo iiPCR method was sensitive enough to detect up to 100 copies of standard DNA template and 10 fg of Fo genomic DNA. In addition, it could directly detect 1 pg of mycelium and 10 spores of Fo without DNA extraction. Our study compared the performance of Fo iiPCR to that of three published in planta molecular detection methods—conventional PCR, SYBR green-based real-time PCR, and hydrolysis probe-based real-time PCR—in field detection of Fo. All diseased field samples yielded positive detection results with high reproducibility when subjected to an Fo iiPCR test combined with a rapid DNA extraction protocol compared to Fo iiPCR with an automated magnetic bead-based DNA extraction protocol. Intraday and interday assays were performed to ensure the stability of this new rapid detection method. The results of detection of Fo in diseased banana pseudostem samples demonstrated that this new rapid detection method was suitable for field diagnosis of Fusarium wilt and had high F1 scores for detection (the harmonic mean of precision and recall of detection) for all asymptomatic and symptomatic Fo-infected banana samples. In addition, banana samples at four growth stages (seedling, vegetative, flowering and fruiting, and harvesting) with mild symptoms also showed positive detection results. These results indicate that this new rapid detection method is a potentially efficient procedure for on-site detection of Fo.

## 1. Introduction

Fusarium wilt (FW) caused by *Fusarium oxysporum* (Fo) is a highly lethal vascular fungal disease that limits crop production worldwide [1,2,3,4]. Fo is the most economically important and commonly encountered species of *Fusarium* [5]. Fo strains attacking the same plant host are generally considered to belong to the same special form, such as bananas (*Musa* spp.), watermelon (*Citrullus lanatus* (Thunb.) Matsum. & Nakai), tomatoes (*Solanum lycopersicum* L.), and lettuce (*Lactuca sativa* L.). Fo is a complex of species consisting of numerous cryptic species, as with other *Fusarium* species complexes, as well as the *F. oxysporum* species complex (FOSC) has suffered from multiple taxonomic/classification systems applied in the past [5].

*Fusarium oxysporum* produces durable thick-walled chlamydospores that are highly tolerant to fungicides, which makes it difficult to eliminate the pathogen [1,6,7,8]. As resting spores, chlamydospores enable Fo to survive in field soil for many years without the presence of hosts [1,2,9,10]. Fo invades its hosts at every stage of their life cycle and colonizes their vascular tissue, leading to the disruption of water transport and wilting [1,9,11]. Therefore, Fo is considered the most damaging species in the *Fusarium* genus [1,11].

To minimize the impact of FW outbreaks, a reliable method for the earlier detection of Fo and diagnosis of FW is required [7,12]. Methods for the efficient diagnosis of crop diseases, especially fungal diseases, in the field help improve disease management. In the specific case of Fo, two essential strategies are employed for reducing the economic impacts of FW [6,12]: first, finding and eradicating any plants infected with Fo at an earlier cultivation stage; and second, avoiding the cultivation of host crops in Fo-contaminated areas. Therefore, a rapid, sensitive, and specific method of detecting Fo has become a crucial strategy for the effective monitoring and control of the pathogen.

Conventional methods of diagnosing FW depend on disease symptoms in samples, morphological observations of isolated pathogens, and further confirmatory diagnosis fulfilling Koch’s postulates [4]. Molecular detection techniques, such as conventional polymerase chain reaction (cPCR) [13], loop-mediated isothermal amplification (LAMP) [14], SYBR Green quantitative PCR (qPCR) [15], hydrolysis probe-based qPCR [16], and real-time LAMP (RealAmp) [17] assays, have been specially developed for Fo diagnosis. Currently, these methods offer high sensitivity and specificity of detection, which makes them more reliable and faster than conventional methods [6,7]. More importantly, these methods can potentially serve as productive tools to monitor the Fo population in plant tissue [13,14,15,16] or soil [17,18]. However, detection methods based on PCR, LAMP, or real-time PCR still require well-trained technicians or expensive instruments [6]. Real-time PCR assays are mainly performed in the laboratory and lack on-site applications [18].

Insulated isothermal PCR (iiPCR) has been successfully used for the detection of *F. oxysporum* f. sp. *cubense* [6] and a variety of other pathogens [19,20,21,22,23,24,25,26,27,28,29,30,31,32,33,34]. Briefly, iiPCR is performed within an inexpensive disposable polycarbonate capillary tube (R-tube^TM^) heated by a constant-temperature heating source for DNA amplification. It is relatively simple and inexpensive, as there is no need for a costly thermocycler [20,21,22,23,24,29,30,35]. Denaturation, annealing, and extension in iiPCR are achieved by natural convection with a single isothermal condition without additional steps [6]. The fluorescence signal of PCR products is detected by an optical sensor module, and signal-to-noise ratio (S/N) detection is used in algorithm-based analysis [29]. The detection of DNA amplicons can be completed automatically by the POCKIT^TM^ analyzer (GeneReach USA, Lexington, MA, USA) with a single default program [22,23,29,30,32].

Our study developed a method using iiPCR assay and a simple DNA preparation protocol for rapid and on-site detection of Fo. Banana FW is a major constraint and serious threat to banana production worldwide [12]. As a case study, we developed a fast detection method using a rapid DNA preparation protocol for the diagnosis of FW in banana plants. The method does not require well-trained technicians for gel electrophoresis of amplicons. Notably, the iiPCR assay for Fo and the rapid, simple DNA preparation protocol can be performed both in the laboratory and on-site. The Fo iiPCR assay has the potential to be a user-friendly method for the routine detection of Fo to reduce its further spread and to ameliorate the impacts of FW.

## 2. Results

### 2.1. Optimization of Fo iiPCR Assay

**Figure 1:**
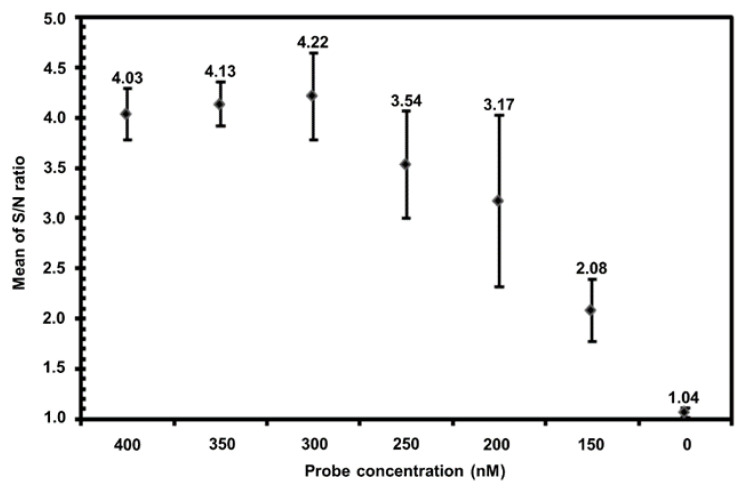
Determination of the optimal probe concentration for insulated isothermal PCR assay of *Fusarium oxysporum* (Fo iiPCR). Different concentrations (ranging from 0 to 400 nM) of the iipFo-1 probe were used for Fo iiPCR. The mean optical fluorescent signals (signal-to-noise (S/N) ratios) of each reaction recorded by the POCKIT™ analyzer at a wavelength of 520 nm were calculated and plotted against each input concentration of the probe. Each error bar denotes the standard deviation of the mean of seven replicate reactions.

The Fn_327_ marker, originally designed by comparing the ITS sequences of 55 different Fusarium sequences [18], was shown to be specific to pathogenic Fo [11,36]. All primers used in this study target the rDNA locus. The Fo-specific primer LNHFnF-1/LNHFnR-1 and the minor groove binder (MGB) hydrolysis probe iipFo-1, established for the sequence of the Fo-specific marker Fn_327_, were used for the Fo iiPCR assay. Concentrations of the iipFo-1 probe were determined to optimize the iiPCR protocol. The results demonstrated that the optimal iipFo-1 probe concentration for the Fo iiPCR reaction was 300 nM with an S/N ratio of 4.22 (standard deviation = 0.43) (Figure 1).

### 2.2. Sensitivity and Specificity Evaluations of the Fo iiPCR Assay

The adaptability of Fo iiPCR for Fo detection was evaluated with the isolates listed in Table 1. Plant pathogenic isolates obtained from several crops were analyzed to assess the specificity of the Fo iiPCR assay. The results were positive for all tested Fo isolates and negative for all other non-Fo pathogens (Table 1). The Fo iiPCR results matched those of the cPCR-based method with the FnSc-1/FnSc-2 primers (Table 1). At the same time, the expected ≈500 bp DNA bands of all isolates were amplified using the template-loading control primer ITS1/ITS4. In addition, rDNA sequence identification with the primers ITS1/ITS4 (Table 1) using the dideoxy chain termination method supported the results of the Fo iiPCR assay.

**Figure 2:**
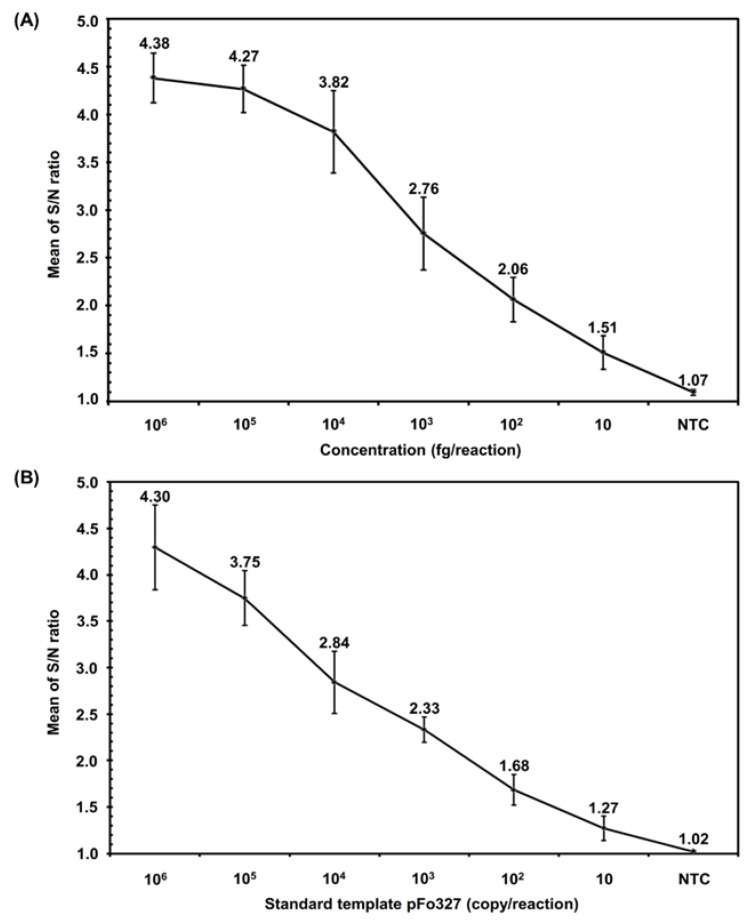
Sensitivity evaluation of the insulated isothermal PCR assay of *Fusarium oxysporum* (Fo iiPCR). Serial dilutions of (**A**) *F. oxysporum* genomic DNA and (**B**) standard DNA were subjected to an iiPCR assay. The mean optical fluorescent signals (signal-to-noise (S/N) ratios) of each reaction reported by the POCKIT™ analyzer at a wavelength of 520 nm were calculated and plotted against each input amount of genomic DNA and standard DNA. Each error bar denotes the standard deviation of the mean of seven replicate reactions.

**Figure 3:**
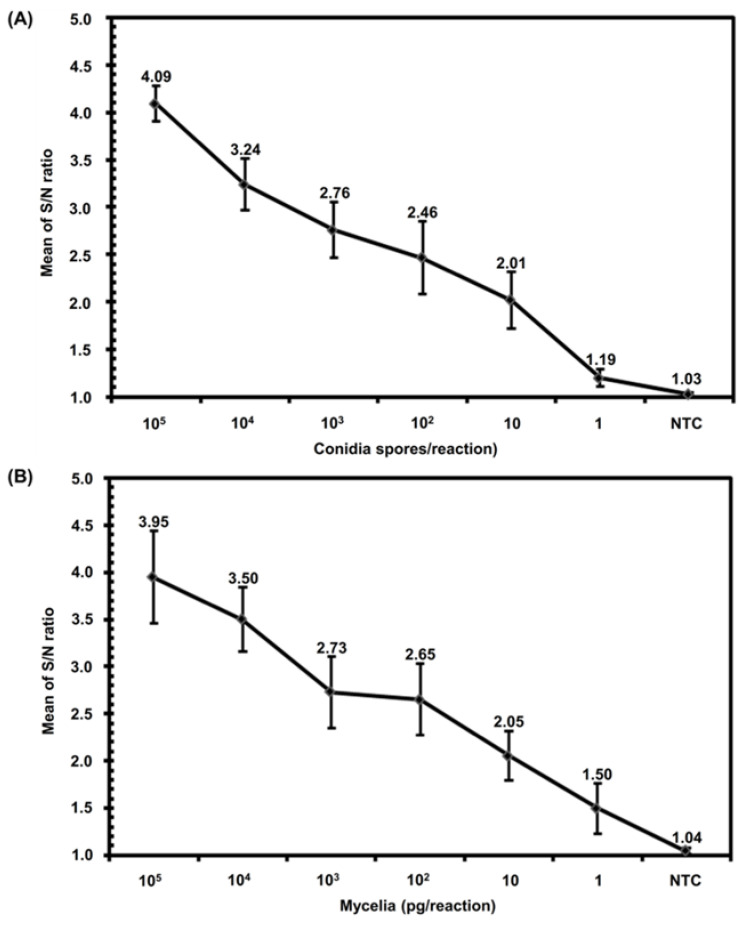
Direct detection of Fusarium oxysporum by insulated isothermal polymerase chain reaction assay (Fo iiPCR). (**A**) Conidia (counted with a hemacytometer under an Axioskop 2 plus microscope, ranged from 1 to 105 spores per reaction) and (**B**) mycelia (ranged from 1 to 105 pg per reaction) of Fo without DNA extraction were subjected to an iiPCR assay. The mean optical fluorescent signals (signal-to-noise (S/N) ratios) of each reaction recorded by the POCKIT™ analyzer at a wavelength of 520 nm were calculated and plotted against each input amount of conidia and mycelia. Each error bar denotes the standard deviation of the mean of seven replicate reactions.

After confirming the specificity of Fo iiPCR, we used different Fo samples (genomic DNA, standard DNA, mycelia, and conidia) to determine their iiPCR sensitivity. The Fo iiPCR results showed that Fo gDNA (ranging from 10^6^ to 10 fg) and Fo standard DNA template pFo_327_ (ranging from 10^6^ to 10^2^ copies) yielded positive results with average S/N ratios of 4.38 ± 0.26 to 1.51 ± 0.17 (Figure 2A) and 4.30 ± 0.46 to 1.68 ± 0.18, respectively (Figure 2B). The results indicated that we obtained significantly positive results with reproducibility even with a small amount of Fo gDNA (10 fg) and standard DNA template pFo_327_ (100 copies) as PCR templates. In addition, as few as 10 conidial spores (Figure 3A) and as little as 1 pg of mycelia (Figure 3B) could be directly detected by this Fo iiPCR assay without any DNA extraction.

### 2.3. Comparison of the Experimental Variability of the Molecular Detection Systems in Field Detection

To assess the reproducibility of the molecular detection methods, duplicate PCR amplification of a banana sample with mild symptoms (intraday) and another duplicate PCR amplification of the same DNA sample (interday) were performed. The digital values of Fo cPCR (transformed from electrophoresis gel images), the Ct values of Fo SYBR-qPCR and Fo Probe-qPCR, and the S/N ratios of Fo iiPCR were then used to calculate the average coefficient of variation (CV) ratios (Table 2). In the detection experiment (field experiment 1), a total of 138 field samples (108 Fo-infected symptomatic samples and 30 Fo-infected but asymptomatic samples) from six different fields were randomly collected for the molecular detection assays. An automated DNA extraction protocol (taco™ mini, Gene-Reach, USA) was used to reduce personal error, and the plate-out and further molecular detection of isolated pathogen (MDIP) assays were used to confirm that the 108 symptomatic and the 30 symptomless banana pseudostems were Fo-infected testers. As shown in Table 2, all experimental variations in Fo iiPCR for the diagnosis of FW in all banana samples were similar and acceptable (average CV ratios ≤ 25% [38]). More importantly, the experimental reproducibility for all banana samples when using Fo iiPCR were similar to that for Fo SYBR-qPCR and Fo Probe-qPCR and significantly higher (lower average CV ratios) than the results obtained with Fo cPCR (Table 3). In all percentage data, the lowest variability in detecting FW was when the Fo SYBR-qPCR and Fo Probe-qPCR assays were used (Table 2).

The detectable rate of Fo iiPCR was also evaluated with the population of 138 samples. In the experiment (field experiment 1), all 108 symptomatic samples gave positive Fo iiPCR results (Table 3), and the results of Fo iiPCR agreed with those of the MDIP assay. In addition, the detection rates of Fo iiPCR, Fo cPCR, Fo SYBR-qPCR, and Fo Probe-qPCR were 100% (30/30), 66.7% (20/30), 100% (30/30), and 100% (30/30), respectively, when the 30 asymptomatic samples were used as testers (Table 3). These results suggest that Fo iiPCR was suitable for the field detection of Fo in infected samples even when they were asymptomatic. The detectable rate of the Fo iiPCR assay was comparable to those of previously used methods for in planta detection of FW. Moreover, the in-field diagnostic results of iiPCR were supported by the symptom characteristics and MDIP results as well as by the result of the Fo SYBR-qPCR and Fo Probe-qPCR assays (Table 3).

### 2.4. Field Detection by Fo iiPCR Assay of FW in Banana Plants at Different Growth Stages

The suitability of the Fo iiPCR assay for detecting FW in banana plants at different growth stages was evaluated. For this purpose, another field experiment (field experiment 2) was performed to assess the reproducibility and applicability of the Fo iiPCR test for field diagnosis of FW on banana plants at seedling, vegetative, flowering and fruiting, and harvesting stages. In field experiment 2, 40 infected (confirmed by MDIP) banana pseudostems at the different growth stages and showing mild symptoms were used as testers. An automated DNA extraction protocol was used to reduce personal error. All testers gave positive results in Fo iiPCR, Fo SYBR-qPCR, and Fo Probe-qPCR assays (Table 4). In addition, all results of the reproducibility evaluation of the three molecular assays were not significant when the 40 banana plants at different growth stages were used as testers (Table 5). These results suggest that samples of banana plants at different growth stages do not reduce the detection rate (recall rate) of Fo iiPCR; therefore, this assay is suitable for detecting Fo in all growth stages of infected samples with mild symptoms.

### 2.5. Rapid Field Detection of FW Using Fo iiPCR Assay and a Simple DNA Preparation Protocol

As a case study, we developed a fast detection method using a rapid DNA preparation protocol for the diagnosis of FW in banana plants. The results of this protocol and Fo iiPCR assay were compared with those of Fo cPCR, Fo SYBR-qPCR, and Fo Probe-qPCR assays for diagnosing FW on banana plants. To be certain of the applicability of the molecular diagnosis systems, two field experiments (3 and 4) were performed. Banana plants at the vegetative stage (plant height of more than 1.5 m and without flowering) with different disease grades and banana plants at different age stages with mild symptoms were used as testers in field experiments 3 and 4, respectively. The four molecular methods (Fo cPCR, Fo SYBR-qPCR, Fo Probe-qPCR, and Fo iiPCR assays, all using a rapid DNA preparation protocol) were validated for the rapid detection of Fo in 48 vegetative banana plants with different disease grades in field experiment 3 and 49 mildly symptomatic banana plants at the four growth stages in field experiment 4.

In field experiment 3, the 48 samples produced positive results in the three molecular assays (Fo SYBR-qPCR, Fo Probe-qPCR, and Fo iiPCR assays); however, the detection rate (recall rate) of FW on the symptomatic banana plants was only 75% (9/12) for Fo cPCR (Table 6). This sample population was also used to assess the reproducibility of the four detection methods. As shown in Table 7, the experimental variability of the three molecular detection assays (Fo SYBR-qPCR, Fo Probe-qPCR, and Fo iiPCR) for the symptomless and symptomatic samples was not significant and was acceptable (average CV ratios ≤ 25%, [16]). The results suggested that the Fo iiPCR assay combined with the rapid DNA preparation protocol has high stability in FW detection, and it is suitable for the field diagnosis of FW on vegetative banana plants. The high recall rates of the different disease grades also demonstrated that this new rapid detection method is suitable for molecular diagnosis of FW on banana plants in the field.

In field experiment 4, the detection rates were 100% for the three molecular assays (Fo SYBR-qPCR, Fo Probe-qPCR, and Fo iiPCR assays) using 49 banana plants at four growth stages as testers (Table 8). When using Fo cPCR for detecting the 49 samples, the detection rates for the two important banana age stages of seedling and vegetative plant without flowering were only 75%, which were worse than the rates obtained with the three molecular assays (Table 8). In addition, the results of the reproducibility evaluation of Fo cPCR were also worse than those of the other three molecular assays (Table 9); all coefficients of variation of the three molecular assays were not considered significant (Tukey HSD test, *p* < 0.05), even though the results of the Fo iiPCR assay were worse than those of the Fo SYBR-qPCR and Fo Probe-qPCR assays (Table 9). These results indicated that the new rapid detection method has the potential to be efficient for the rapid screening of Fo-infected banana plants of different ages.

In the four field experiments, the precision (True positives/True positives + False positives), recall (True positives/True positives + False negatives), accuracy ((True positives + True negatives)/(True positives + False positives + False negatives + True negatives)), and F1 scores (the harmonic mean of precision and recall) of the eight molecular detection protocols were used for comparisons. As shown in Table 10, when using the three high-stability molecular detection assays (Fo SYBR-qPCR, Fo Probe-qPCR, and Fo iiPCR) for detecting infection in both asymptomatic and symptomatic banana samples, the recall and accuracy of the six protocols were better than those of the other two Fo cPCR-protocols, which suggested that use of the rapid DNA extraction–iiPCR protocol allowed accurate detection without false negatives in molecular diagnosis of FW on banana plants in the field. In addition, higher F1 scores of the three high-stability molecular detection assays also supported the conclusion that the rapid DNA extraction–iiPCR protocol was suitable for the detection of FW in field-infected banana samples even when asymptomatic (Table 10). It is worth noting that the samples of infected but asymptomatic banana pseudostems yielded worse F1 scores when Fo cPCR was used with automated DNA extraction or rapid DNA extraction (Table 10), indicating that the two Fo cPCR protocols were not suitable for field diagnosis of FW in asymptomatic banana samples.

## 3. Discussion

*Fusarium oxysporum* is regarded as one of the top five fungal plant pathogens in the world, and it can infect more than 100 different hosts [39]. Economically, this pathogen is responsible for the devastating FW on various crops and causes severe crops losses [40]. However, Fo has not been easy to control with fungicides in the field [41,42]. The prevention of the spread of Fo from infected to uninfected plants as much as possible is the top priority in the management of FW [6,7].

The ITS marker Fn_327_, which was originally designed by Zhang et al. [18], can be used to specifically tag *F. oxysporum* f. sp. *niveum* with the primers Fn-1/Fn-2 [18] or to tag pathogenic Fo with the primers FnSc-1/FnSc-2 [11] and LNHFnF-1/LNHFnR-1 [18]. In the search for a newer and faster way to detect Fo, this study developed a hydrolysis probe-based Fo iiPCR assay using the primers LNHFnF-1/LNHFnR-1 and probe iipFo-1 based on the Fn_327_ sequence. The specificity of Fn_327_ was confirmed by testing with 72 isolates of *F. oxysporum* [11,36]. In this study, the specificity evaluation results of Fo iiPCR were matched with the results of the cPCR-based method and supported by the data from dideoxy chain termination sequencing of rDNA (Table 1). Van Dam et al. [43] indicated that the *F. oxysporum* genome contains approximately 100 copies of rDNA. This supports the sensitivity of this iiPCR, which was 100 copies of pFo_327_ (Figure 2B). In addition to detection with purified DNA, the Fo iiPCR assay can directly detect 1 pg of mycelium and 10 spores of Fo without DNA extraction (Figure 3), which indicates that it has potential for use in the rapid screening of pure cultures of Fo. In our results, no amplicons of Fn_327_ were obtained from the Fo endophytes. However, degenerated virulence, irregular development, and polyphyletic origin are common features for Fo [22]. This risk may mitigate the widespread use of this locus for pathogen detection. We will perform the risk investigation for the developed method in the near future. Previous studies have reported some applications of qPCR methods [15,16], LAMP [14], and RealAmp [17] for the efficient detection of Fo. Our evaluation of the sensitivity of Fo iiPCR showed that it was comparable to previously published methods. Specifically, Fo iiPCR, SYBR Green real-time PCR, and LAMP generate a positive signal with as little Fo genomic DNA as 1 fg, 1 fg, and 10 fg, respectively. The detection sensitivity of hydrolysis probe-based real-time PCR was 10 fg of standard DNA [16]. The sensitivity of RealAmp and this iiPCR was 3.82 × 10^3^ [17] and 10^2^ copies of standard DNA, respectively.

The Fo iiPCR assay was used for the rapid in planta detection of Fo. Traditional methods for diagnosing plant diseases based on plate-out assay and Koch’s postulates are time consuming [43], as are standard procedures such as gel electrophoresis for analyzing PCR products in the PCR-based detection method (e.g., the Fo cPCR assay). Therefore, the Fo iiPCR assay should be able to address the shortcomings of these protocols. The other two Fo detection methods—Fo SYBR-qPCR and Fo Probe-qPCR assays—still require relatively expensive instruments and well-trained technicians for data analysis. In comparison, the Fo iiPCR assay can be performed with the relatively simple iiPCR device (POCKIT™ Nucleic Acid Analyzer) that can detect PCR amplicons using a module combining an optical sensor and S/R detection as well automatically convert detection results to “+” or “−” shown on its display screen. Overall, the major advantage of Fo iiPCR is that it enables compact PCR reaction and signal detection within one machine. Unlike Fo SYBR-qPCR and Fo Probe-qPCR, it automatically simplifies the DNA amplification procedure and amplicon detection with the use of a single default program of the iiPCR device without further data analysis. The major disadvantage of Fo iiPCR is that it may not be as stable as qPCR (even if the reproducibility evaluation indicated that the results of the three assays were not significantly different (Table 2 and Table 5)) and is not as quantitative as the qPCR methods [22].

A higher level of variability indicates poorer reproducibility [44,45]. We used an automated DNA extraction protocol and the symptomatic pseudostems as samples, and we observed the highest CVs with the Fo cPCR assay, which indicated the worst reproducibility (Table 2). In contrast, Fo SYBR-qPCR and Fo Probe-qPCR demonstrated the lowest CVs when symptomatic pseudostems were used for Fo detection. When the procedures were applied to symptomless pseudostems (Table 2) and all age stages of banana plants (seedling, vegetative, flowering and fruiting, and harvesting) (Table 5), both Fo SYBR-qPCR and Fo Probe-qPCR assays demonstrated the lowest CVs, indicating that the two assays have the highest experimental reproducibility. When Fo cPCR was used with mildly symptomatic/symptomless samples, the results of interday and intraday assays were not as good at 18.10–39.96% and 20.66–59.65%, respectively (Table 2), indicating that the assay was not suitable for diagnosing such samples. Furthermore, when using Fo iiPCR, the variability of the test results of the reproducibility assays for vegetative banana plants of different disease grades and mildly symptomatic banana plants at all age stages was 2.99–5.43% (Table 2) and 2.45–4.72% (Table 5), respectively, indicating that Fo iiPCR is suitable for detecting FW on banana. When using Fo iiPCR to detect FW on symptomless pseudostems, the variability of the results of the interday and intraday assays was 5.43% and 5.37%, respectively (Table 2) and the detection rate for the tester population was 100% (Table 3). Therefore, we can conclude that the Fo iiPCR results for FW detection, in the case of banana plants, are reproducible. Overall, Fo SYBR-qPCR and Fo Probe-qPCR are the most stable methods of detecting Fo infection in all banana samples, since they had the highest reproducibility.

The precision, recall, accuracy, and F1 scores of Fo iiPCR were evaluated in four field experiments. A total of 275 testers including 186 vegetative banana plants with different disease grades were used in molecular detections to evaluate the Fo iiPCR assay’s adaptability in in planta clinical detection of FW. Recall is vitally important in detection assays, and high field detection recall (recall = True positives/(True positives + False negatives)) of Fo by the Fo iiPCR assay was observed for the results yielded from the 275 field-collected samples (Table 10). It is worth noting that the patterns of detection of FW (including precision, recall, accuracy, and F1 scores) by Fo iiPCR did not differ between the two DNA extraction protocols (Table 10). The results of detection using Fo iiPCR were also supported by the results of the MDIP assay and the in planta detection assays, Fo SYBR-qPCR and Fo Probe-qPCR. These results showed that although the reproducibility of the Fo iiPCR results was worse than the Fo qPCR results, the detection recall results of the four field detection methods were not affected (Table 10). In addition, the detection pattern of the rapid DNA extraction protocol was comparable to that of the automated DNA extraction protocol for all samples (Table 10). These data supported the conclusion that the new rapid detection method was suitable for molecular diagnosis of FW in banana samples with different symptoms and at different growth stages. The time needed for each of the molecular detection assays (including DNA amplification and signal detection) was as follows: for cPCR, c.a. 3 h [37]; for SYBR-qPCR, c.a. within 2 h [37]; for Probe-qPCR, c.a. within 2 h [37]; and for iiPCR developed in this study, 60 min. In addition, a simple potable iiPCR device (POCKIT™ Micro Nucleic Acid Analyzer, GeneReach USA, MA, USA) is available, and the Fo iiPCR assay requires less time (within 30 min) than all the other assays, indicating that it is likely to be an effective molecular tool for on-site detection of FW.

In this study, all 275 testers were confirmed as Fo-infected samples by the MDIP assay in the comparison of all detection protocols used and the precision of values, recall, accuracy, and F1 score. However, the MDIP assay was unable to detect all asymptomatic plants in FW-outbreak fields. In the future, we will perform a detection experiment to determine whether molecular detection methods based on Fo qPCR and Fo iiPCR are suitable for the diagnosis of FW in banana plants with latent infection.

## 4. Materials and Methods

### 4.1. Pathogens and Growth Conditions

The Fo isolates confirmed by a pathogenicity test of their original hosts are listed in Table 1. Other fungal pathogens, including *F. verticillioides* (YHL-F056), *Rhizoctonia solani* (SMS-F012 and SMS-F013), and *Colletotrichum orbiculare* (LLH-F001), were used for comparison (Table 1). Each tested Fo isolate was cultured from a single spore on a peptone pentachloronitrobenzene (PCNB) agar medium (1.5% peptone, 0.1% PCNB, 2% agar, 0.1% KH_2_PO_4_, 0.05% MgSO_4_·7H_2_O, 0.1% neomycin, and 0.03% streptomycin) [13,46]. The mycelium of *R. solani* and single spores of other fungi were cultured on a PDA medium. After seven days of incubation, mycelia were freeze-dried for genomic DNA (gDNA) extraction.

### 4.2. Primer and Hydrolysis Probe Design

The Fn_327_ marker amplified by the specific primers FnSc-1/FnSc-2 (nt1-26/nt302-327) was shown to be specific to Fo in a previous study [11,36]. A minor groove binder (MGB) hydrolysis probe iipFo-1 (5′-6-FAM-GTAACTTCTGAGTAAAACC-MGB-NFQ-3′) and an Fo-specific primer LNHFnF-1/LNHFnR-1 (nt53-72/nt134-153) were designed for the Fo iiPCR assay according to the sequence of Fn_327_ [11,36]. The primers FnSc-1/FnSc-2 and LNHFnF-1/LNHFnR-1 have previously been used for Fo detection with PCR [11] and qPCR [36]. The ≈500-bp rDNA region was amplified by conserved primers ITS1/ITS4 [37]. The ITS1/ITS4 amplicons were used for identifying the isolates tested and as template-loading controls of PCR for molecular detection assays in this study. Another PCR-based molecular assay using the primers FnSc-1/FnSc-2 [11] was used to double-check the specificity results of the Fo iiPCR assay. The primer sequences are listed in Table 11. The PCR conditions and protocols used for primers ITS1/ITS4, FnSc-1/FnSc-2, and LNHFnF-1/LNHFnR-1 were according to White et al. [47], Lin et al. [11], and Huang et al. [36], respectively.

### 4.3. Sample Preparation for Sensitivity Evaluation of Fo iiPCR

Four testers—gDNA, standard DNA, mycelia, and conidia—were used in sensitivity assays of Fo iiPCR. The gDNA was extracted according to Lin et al. [6,11,13,15] and dissolved in a 0.1× TE buffer (pH 8.0). A 327-bp DNA sequence was amplified by the primers FnSc-1/FnSc-2. The gel-purified amplicons were cloned into pGEM^®^-T Easy vector (Promega, WI, USA), sequenced, and used as the standard DNA template (named pFo_327_) for iiPCR. The plasmid pFo_327_ was linearized by cleaving it outside the insertion with the restriction enzyme *Eco*RI. The copy number concentration of the linearized pFo_327_ was determined with a spectrophotometer (NanoDrop Lite, Thermo Fisher Scientific, OH, USA). The standard DNA pFo_327_ dissolved in a 0.1× TE buffer was used for further Fo iiPCR assays. Conidial spores were counted with a hemacytometer under a microscope (Axioskop 2 plus, Carl Zeiss, Germany). Mycelia of Fo were snap-frozen in liquid nitrogen and ground into fine powder using a mortar and pestle. The conidial and mycelial samples were directly subjected to sensitivity evaluations with Fo iiPCR but without DNA extraction.

### 4.4. Fo iiPCR Assay

Fo iiPCR amplification was carried out in 50-μL R-tube^TM^ tubes containing the test gDNA, 0.5 mM of each primer (LNHFnF-1/LNHFnR-1), 300 nM of iipFo-1 probe (Applied BioSystem, Foster City, CA, USA), 1 U of KAPA Taq DNA polymerase (Kapa Biosystems, Wilmington, MA, USA), and 1× Uni-ii LS Buffer (GeneReach USA, Lexington, MA, USA). The iiPCRs were performed in a POCKIT^TM^ Nucleic Acid Analyzer (GeneReach USA, MA, USA) and completed in 60 min. The POCKIT™ analyzer collected the optical fluorescent signals (signal-to-noise (S/N) ratios) at a wavelength of 520 nm through an image sensor and converted them automatically to “+” or “−” according to the default S/N ratio by the built-in S/N thresholds (≥1.3) algorithm; the signals were then directly shown on the display screen of POCKIT^TM^ Nucleic Acid Analyzer. The data were exported for further analysis.

### 4.5. Specificity Determination and Sensitivity Evaluation

The gDNA of plant pathogens isolated from crops was used for determining the specificity of the Fo iiPCR method. These pathogens are listed in Table 1. For sensitivity assays, gDNA (ranging from 10^6^ to 10 fg), the standard DNA pFo_327_ (ranging from 10^6^ to 10 copies), conidia (drops of the spore suspension counted with a hemacytometer under an Axioskop 2 plus microscope and individually transferred to R-tube^TM^ tubes for further iiPCR; ranging from 10^5^ to 10^0^ spores), and mycelia (ranging from 10^5^ to 10^0^ pg), were subjected to sensitivity tests.

### 4.6. Sampling Criteria for Infected Tissues and MDIP Assay

A previously described molecular method, molecular detection of isolated pathogen (MDIP) [7,13,37], was used to confirm Fo infection of banana pseudostems collected from the field. Specifically, three levels of disease symptoms were recorded: mild (necrosis covering less than one-third of the total area of pseudostem); moderate (necrosis less than two-thirds, but equal to or more than one-third, of the total area), and severe (necrosis equal to or more than two-thirds of the total area). The field-collected banana pseudostems showing these symptoms were individually sampled, surface-sterilized in 1% NaHClO for 1 min, rinsed three times with sterile water, and air-dried in a laminar flow hood to avoid contamination by epiphytic microbes. The surface-sterilized dried pseudostems were then cut into small pieces of approximately 1 cm^2^ and placed on a Nash-PCNB agar medium for pathogen isolation. Simultaneously, the nearby area (0.3 g) of each section of banana pseudostem was used for gDNA extraction. The DNA samples (50 ng) from pathogens (MDIP) and from plant tissue (a combination of plant plus pathogens) were then used in molecular detection assays.

### 4.7. Molecular Detection Assays

To compare the suitability of the Fo iiPCR method in the field, various field detection methods were performed to diagnose FW on banana plants. To reduce personal error, an automated DNA extraction protocol (taco™ mini, GeneReach, USA) was used according to the manufacturer’s instructions. Three previously published in planta molecular detection methods, i.e., Fo cPCR (conventional PCR) [11], Fo SYBR-qPCR (SYBR Green qPCR) [36], and Fo probe-qPCR (hydrolysis probe-based qPCR) assays [36], were also used in the field detection evaluation in order to compare their results with that of the Fo iiPCR assay.

cPCR was carried out in 8-strip PCR tubes (Bio-Rad Laboratories, Hercules, CA, USA). Each 25 μL PCR reaction contained the test gDNA, 0.25 μM of each primer (LNHFnF-1/LNHFnR-1), and 1× KAPA Taq ReadyMix PCR Kit (Kapa Biosystems, MA, USA). The thermocycler program setting for cPCR was as follows: 30 cycles of 94 °C for 30 s (denaturing); 62 °C for 30 s (annealing), and 72 °C for 120 s (polymerizing); with a final cycle extending amplification conditions to 72 °C for 10 min. PCRs were performed in a Thermal Cycler (T100^TM^, Bio-Rad Laboratories, CA, USA). All PCR products were separated by electrophoresis on 2% agarose gel and visualized by Gel DocTM EZ Imager (Bio-Rad Laboratories, CA, USA). In addition, the electrophoresis gel images were transformed into digital results with ImageJ software, according to the protocol described by Antiabong et al. [48], for further reproducibility evaluation.

SYBR-qPCR was carried out in 8-strip PCR tubes (Bio-Rad Laboratories, CA, USA). Each 25 μL qPCR reaction contained the test templates, 0.25 μM of each primer (LNHFnF-1/LNHFnR-1), and 1X KAPA SYBR^®^ FAST qPCR Kit Master Mix Universal (Kapa Biosystems, MA, USA). The thermocycler program setting for SYBR-qPCR was as follows: 40 cycles of 95 °C for 10 s (denaturing); 60 °C for 20 s (annealing and polymerizing); and 60 °C to 97 °C (at 0.1 °C increments every 1 s, with melting curve analysis to verify specificity).

The probe-qPCR assay was carried out in 8-strip PCR tubes (Bio-Rad Laboratories., CA, USA). Each 25 μL qPCR reaction contained the test templates, 0.25 μM of each primer (LNHFnF-1/LNHFnR-1), a Black Hole Quencher 1 (BHQ^®^-1) pLNH probe (5′-FAM-GTAACTTCTGAGTAAAACC-BHQ-3′), and 1X KAPA Probe FAST qPCR Kit Master Mix Universal (Kapa Biosystems, MA, USA). The thermocycler program setting for probe-qPCR was as follows: 40 cycles of 95 °C for 10 s (denaturing) and 60 °C for 20 s (annealing and polymerizing). The standard curves of the two qPCR assays were created automatically with the CFX96 Touch^TM^ Real-Time PCR Detection System (Bio-Rad Laboratories, CA, USA) by plotting the threshold cycle (Ct) value against the target amount of DNA. In this study, all molecular assays in the sensitivity and specificity tests were performed for seven independent replicates in order to enable an assessment of significance.

Two validations—intraday (duplicate PCR amplification of a banana plant sample) and interday (duplicate PCR amplification of the same DNA sample performed at different time points)—were performed according to Skottrup et al. [42] to assess the experimental variability of the molecular detection systems. These intraday (different DNA extracts from the same sample) and interday (parallel PCRs from the same DNA extract) validations were assessed in four independent field experiments by calculating the coefficient of variation (CV) of duplicate PCR amplification of field-infected samples.

### 4.8. Molecular Field Detection Assays

Four field experiments were performed with a total of 275 banana pseudostem samples to assess whether or not the molecular detection assays (Fo cPCR, Fo SYBR-qPCR, Fo Probe-qPCR, and Fo iiPCR) were suitable for detection of FW in plants with varying symptoms (field experiments 1 and 3) and at different ages (field experiments 2 and 4). In field experiments 1 and 3, field samples (including Fo-infected symptomatic samples and Fo-infected but asymptomatic samples) from six different fields were randomly collected for molecular detection assays. In field experiments 2 and 4, banana plant samples at four growth stages—seedling (plant height of less than 1.5 m), vegetative (plant height of more than 1.5 m, but without flowering), flowering and fruiting, and harvesting (approximately two months after flowering)—were used as testers. The DNA of banana pseudostems collected from field experiments 1 and 2 was extracted using an automated DNA extraction protocol (taco™ mini, Gene-Reach, USA) and from field experiments 3 and 4 using a rapid DNA extraction protocol. For rapid DNA extraction, the surface-sterilized banana pseudostems were cut into 1 cm^2^ sections and ground in a mortar with 0.6 mL of lysis buffer (50 mM NaOH). After sample homogenization, pseudostem lysates were mixed with 1.4 mL of TE buffer (10 mM Tris-HCl, 1 mM EDTA, pH 8.0). After rapid DNA extraction, 20 µL of each of the extracts was diluted with 400 µL of ddH_2_O and subjected to further molecular detection. The automated DNA extraction protocol was carried out according to the manufacturer’s instructions (taco™ mini, Gene-Reach, USA). Tester DNA was dissolved in a 0.1 × TE buffer (1 mM Tris–HCl and 0.1 mM EDTA, pH 8.0) and stored at −20 °C for further molecular detection.

## 5. Conclusions

This is a novel study that describes a convenient diagnostic method based on the iiPCR assay for the rapid detection of Fo. This Fo iiPCR assay has high sensitivity to Fo DNA, making it possible to detect samples infected with a low amount of Fo. The Fo iiPCR assay can also be used to directly detect 10 pg of mycelium and 10 spores of Fo without DNA extraction. Our results suggest that Fo iiPCR is a suitable alternative to cPCR and qPCR assays for Fo detection and FW diagnosis. In this study, the Fo iiPCR assay was used for field detection of Fo on banana plants, and it generated results comparable to those of the previously published detection methods Fo SYBR-qPCR and Fo Probe-qPCR. The Fo iiPCR assay was able to automatically simplify DNA amplification and amplicon detection to a single default program of 60 min. The results of detection of Fo in diseased banana pseudostem samples demonstrated that this new rapid detection method was suitable for field diagnosis of FW, with a high F1 score of detection (the harmonic mean of precision and recall of detection), in all asymptomatic and symptomatic vegetative banana samples. In addition, this Fo iiPCR assay was suitable for the detection of Fo in field-infected banana plants of different ages even though the infected plants were only mildly symptomatic. These results confirm that the Fo iiPCR assay is a good candidate to serve as a rapid, specific, and sensitive tool for routine in planta prescreening of Fo. However, degenerated virulence, irregular development and polyphyletic origin are common features for Fo [49]. The FOSC has suffered from multiple taxonomic/classification systems. For these reasons, we still encourage greater due diligence using other assessments/assays before concluding that plants are infected with the specific special form of Fo which causes disease in the host.

## Figures and Tables

**Table 1:** Fungal isolates of the phytopathogens and the results of their detection with the molecular detection methods used in this study.

Isolate Code Number	Diseases/Predict Species (Complex)	Original Host/Tissue	Geographic Locations	PCR-Based Identification Methods Used in the Study		
				ITS1/ITS4 ^a^	FnSc-1/FnSc-2 ^b^	Fo iiPCR ^c^
ATCC38741	Fusarium wilt (FW)/*Fusarium oxysporum* (*Fusarium oxysporum* species complex, FOSC)	Banana (*Musa* sp.)/Pseudostem (P)	Taiwan	+	+	+
ATCC76243	FW/*Fusarium oxysporum* (FOSC)	Banana/P	Queensland, Australia	+	+	+
ATCC 76257	FW/*Fusarium oxysporum* (FOSC)	Banana/P	Honduras	+	+	+
ATCC 76262	FW/*Fusarium oxysporum* (FOSC)	Banana/P	Taiwan	+	+	+
ATCC96285	FW/*Fusarium oxysporum* (FOSC)	Banana/P	Southeastern Queensland, Australia	+	+	+
ATCC96290	FW/*Fusarium oxysporum* (FOSC)	Banana/P	Southeastern Queensland, Australia	+	+	+
YHL-F015	FW/*Fusarium oxysporum* (FOSC)	Banana/P	Pingtung, Taiwan	+	+	+
YJL-F044	FW/*Fusarium oxysporum* (FOSC)	Banana/P	Pingtung, Taiwan	+	+	+
YJL-F068	FW/*Fusarium oxysporum* (FOSC)	Banana/P	Taichung, Taiwan	+	+	+
YHL-F006	FW/*Fusarium oxysporum* (FOSC)	Bitter gourd (*Momordica charantia* L.)/Stem (S)	Kaohsiung, Taiwan	+	+	+
YHL-F002	FW/*Fusarium oxysporum* (FOSC)	Chickpea (*Cicer arietinum* L.)/S	Pingtung, Taiwan	+	+	+
YHL-F003	FW/*Fusarium oxysporum* (FOSC)	Cowpea (*Vigna sesquipedalis* L.)/S	Changhua, Taiwan	+	+	+
GFH-F009	FW/*Fusarium oxysporum* (FOSC)	Cucumber (*Cucumis sativus* L.)/S	Pingtung, Taiwan	+	+	+
YHL-F019	FW/*Fusarium oxysporum* (FOSC)	Gladiolus *(Gladiolus psittacinus* L.)/S	Taichung, Taiwan	+	+	+
ATCC76616	FW/*Fusarium oxysporum* (FOSC)	Lettuce (*Lactuca sativa* L.)/S	California, USA	+	+	+
YHL-F021	FW/*Fusarium oxysporum* (FOSC)	Lettuce/S	Taitung, Taiwan	+	+	+
YHL-F035	Basal rot/*Fusarium oxysporum* (FOSC)	Lily *(Lilium* sp.)/Root (R)	Taoyuan, Taiwan	+	+	+
YHL-F038	FW/*Fusarium oxysporum* (FOSC)	Loofah *(Luffa cylindrica* L.)/P	Nantou, Taiwan	+	+	+
TDC-F009	FW/*Fusarium oxysporum* (FOSC)	Melon (*Cucumis melo* L.)/S	Kaohsiung, Taiwan	+	+	+
YHL-F040	FW/*Fusarium oxysporum* (FOSC)	Onion (*Allium cepa*)/S	Taichung, Taiwan	+	+	+
YHL-F041	FW/*Fusarium oxysporum* (FOSC)	Radish (*Raphanus sativus* L.)/R	Nantou, Taiwan	+	+	+
DWH1	FW/*Fusarium oxysporum* (FOSC)	Strawberry (*Fragaria* × *ananassa*)/S	Miaoli, Taiwan	+	+	+
YHL-F042	FW/*Fusarium oxysporum* (FOSC)	Tomato (*Solanum lycopersicum*)/S	Taichung, Taiwan	+	+	+
ATCC18467	FW/*Fusarium oxysporum* (FOSC)	Watermelon (*Citrullus lanatus* Thunb.)/S	South Carolina, USA	+	+	+
ATCC62940	FW/*Fusarium oxysporum* (FOSC)	Watermelon/Seed	Texas, USA	+	+	+
YHL-F018	Fusarium blight/*F. acuminatum* (*Fusarium tricinctum* species complex)	Bermuda Grass (*Cynodon dactylon* L.)/S	Taoyuan, Taiwan	+	−	−
PFW-F11	Wilt/*F. lateritium* (*Fusarium lateritium* species complex)	Coffee (*Coffea* sp.)/S	Taoyuan, Taiwan	+	−	−
PFW-L10	Wilt/*F. xylarioides* (*Fusarium fujikuroi* species complex, FFSC)	Coffee/S	Taoyuan, Taiwan	+	−	−
YHL-F056	Panicle rot/*F. verticillioides* (FFSC)	Rice (*Oryza sativa* L.)/Panicle	Pingtung, Taiwan	+	−	−
YJL-F108	Alternaria speckle/*A. alternata*	Banana/Leaf (L)	Pingtung, Taiwan	+	−	−
YJL-F115	Anthracnose/*Colletotrichum gloeosporioides*	Banana/L	Pingtung, Taiwan	+	−	−
YJL-F150	Cordana leaf spot/*Neocordana musae*	Banana/L	Pingtung, Taiwan	+	−	−
YJL-F119	Deightoniella leaf spot/*Deightoniella torulosa*	Banana/L	Pingtung, Taiwan	+	−	−
CJW-F024	Anthracnose/*Colletotrichum* sp.	Cocoa (*Theobroma cacao*)/L	Pingtung, Taiwan	+	−	−
PFW-L3	Anthracnose/*C. theobromicola*	Coffee/L	Taoyuan, Taiwan	+	−	−
LLH-F001	Anthracnose/*C. orbiculare*	Cucumber/L	Tainan, Taiwan	+	−	−
CJW-F026	Anthracnose/*Colletotrichum* sp.	Eggplant (*Solanum melongena*)/Fruit (F)	Pingtung, Taiwan	+	−	−
JSH-F005	Phytophthora blight/*Phytophthora capsici*	Eggplant/F	Pingtung, Taiwan	+	−	−
CJW-F011	Anthracnose/*C. gloeosporioides*	Guava (*Psidium guajava* L.)/F	Pingtung, Taiwan	+	−	−
SSH-F096	Guava scab/*Neopestalotiopsis* sp.	Guava/F	Kaohsiung, Taiwan	+	−	−
CJW-F0025	Anthracnose/*Colletotrichum* sp.	Jujube (*Ziziphus mauritiana*)/F	Pingtung, Taiwan	+	−	−
CJW-F0012	Anthracnose/*C. gloeosporioides*	Mango (*Mangifera indica*)/F	Pingtung, Taiwan	+	−	−
TDC-F016	Fruit rot/*Alternaria* sp.	Melon/F	Kaohsiung, Taiwan	+	−	−
TDC-F013	Anthracnose/*C. gloeosporioides*	Melon/F	Kaohsiung, Taiwan	+	−	−
JSH-F025	Anthracnose/*C. gloeosporioides*	Papaya (*Carica papaya*)/F	Tainan, Taiwan	+	−	−
ZWY-F002	Black spot/*Cercospora cydoniae*	Papaya/L	Pingtung, Taiwan	+	−	−
CJW-F033	Phytophthora blight/*Phytophthora palinuora*	Papaya/F	Pingtung, Taiwan	+	−	−
JSH-F004	Leaf spot/*A. alternata*	Papaya/L	Pingtung, Taiwan	+	−	−
SMS-F012	Sheath blight (SB)/*Rhizoctonia solani*	Rice/Leaf sheath	Pingtung, Taiwan	+	−	−
824	Rice blast/*Pyricularia oryzae*	Rice/L	Pingtung, Taiwan	+	−	−
PTS-F012	Lasiodiplodia rot/*Lasiodiplodia pseudotheobromae*	Soybean (*Glycine max*)/S	Pingtung, Taiwan	+	−	−
PTS-F021	Lasiodiplodia rot/*L. theobromae*	Soybean/S	Pingtung, Taiwan	+	−	−
PTS-F031	Lasiodiplodia rot/*L. iranensis*	Soybean/S	Pingtung, Taiwan	+	−	−
PTS-F022	Charcoal rot/*Macrophomina phaseolina*	Soybean/S	Pingtung, Taiwan	+	−	−
CHY-F001	Gray mold/*Botrytis cinerea*	Strawberry/F	Pingtung, Taiwan	+	−	−
ML123	Anthracnose/*C. gloeosporioides*	Strawberry/F	Miaoli, Taiwan	+	−	−
SSH-F142	Pestalotia leaf spot/*Pestalotia longiseta*	Strawberry/L	Pingtung, Taiwan	+	−	−
M0029	Anthracnose/*C. camelliae*	Tea (*Camellia sinensis* L.)/L	Taoyuan, Taiwan	+	−	−
T0013	Gray blight/*Pseudopestalotiopsis theae*	Tea/L	Taoyuan, Taiwan	+	−	−
PJH-F048	Early blight/*A. alternata*	Tomato/L	Pingtung, Taiwan	+	−	−
PP151	Leaf spot/*A. solani*	Tomato/L	Pingtung, Taiwan	+	−	−
PP086	Anthracnose/*C. gloeosporioides*	Tomato/F	Pingtung, Taiwan	+	−	−
PP102	Target leaf spot/*Corynespora cassiicola*	Tomato/L	Pingtung, Taiwan	+	−	−
YHL-F061	Endophyte/*F. oxysporum*	Banana/P	Pingtung, Taiwan	+	−	−
JYY-F068	Endophyte/*F. oxysporum*	Banana/P	Pingtung, Taiwan	+	−	−
JYY-F073	Endophyte/*F. oxysporum*	Banana/L	Pingtung, Taiwan	+	−	−
LNH-F104	Endophyte/*F. oxysporum*	Strawberry/L	Miaoli, Taiwan	+	−	−
CJW-F038	Endophyte/*F. oxysporum*	Tomato/L	Pingtung, Taiwan	+	−	−

^a^ ITS1/ITS4 primers [37] used to amplify and sequence a ≈500-bp rDNA region by the dideoxy chain termination method supported the results of the Fo iiPCR assay for identifying the isolates tested; ITS1/ITS4 amplicons were also used as template-loading controls of PCR for molecular detection assays in this study. ^b^ The Fn_327_ marker amplified by the specific primers FnSc-1/FnSc-2 (nt1-26/nt302-327) was shown to be specific to *Fusarium oxysporum* in an earlier study [11,36]. ^c^ The Fo iiPCR results were matched with those of the conventional PCR-based method through the primers FnSc-1/FnSc-2.

**Table 2:** Evaluation of reproducibility of the molecular detection assays using various *Fusarium oxysporum*-infected asymptomatic and symptomatic samples as testers.

Infected Samples ^a^	Reproducibility Assay (Coefficient of Variation (CV), %) ^b^							
	cPCR		SYBR-qPCR		Probe-qPCR		iiPCR	
	Intraday	Interday	Intraday	Interday	Intraday	Interday	Intraday	Interday
Symptomless samples	39.96 ± 8.68 b	59.65 ± 17.88 a	1.41 ± 0.46 f	1.55 ± 0.66 f	2.08 ± 0.81 ef	1.62 ± 0.47 f	5.37 ± 2.88 cdef	5.43 ± 2.15 cdef
Mild symptom samples	18.10 ± 8.73 cd	20.66 ± 8.58 cd	0.54 ± 0.11 f	0.75 ± 0.14 f	0.47 ± 0.21 f	0.43 ± 0.22 f	4.31 ± 1.39 def	2.99 ± 0.29 def
Moderate symptom samples	17.66 ± 6.58 cde	17.31 ± 6.66 c	0.75 ± 0.22 f	0.53 ± 0.07 f	0.37 ± 0.06 f	0.54 ± 0.14 f	3.38 ± 1.58 def	3.74 ± 1.66 def
Severe symptom samples	16.65 ± 6.00 ab	19.42 ± 9.20 b	0.93 ± 0.17 f	0.64 ± 0.16 f	0.56 ± 0.20 ef	0.64 ± 0.20 f	4.06 ± 0.58 def	3.87 ± 1.32 cdef

^a^ Banana pseudostems with mild (necrosis covering less than one-third of the total area of pseudostem), moderate (less than two-thirds but equal to or more than one-third of the total area of pseudostem), or severe (equal to or more than two-thirds of the total area of pseudostem) infection. ^b^ Coefficient of variation (CV) is calculated as standard deviation divided by the mean of each assay (n = 138) × 100%. R studio (RStudio, Inc., Vienna, Austria) with the stats package was used to conduct the statistical analyses. All percentage data were subjected to ANOVA, and Tukey’s honestly significant difference (HSD) test was then performed (means indicated with different letters are significantly different at the *p* < 0.05 level).

**Table 3:** Comparison of Fo iiPCR and molecular methods for field detection of various *Fusarium oxysporum*-infected asymptomatic and symptomatic samples using an automated DNA extraction protocol.

Infected Samples ^a^	MDIP Assay ^b^	In Planta Detection ^c^				
		cPCR	SYBR-qPCR	Probe-qPCR	iiPCR	
		Detection Rate (%)	Detection Rate (%)	Detection Rate (%)	Detection Rate (%)	S/N Ratio
Symptomless samples	30/30 (100)	20/30 (66)	30/30 (100)	30/30 (100)	30/30 (100)	1.363 ± 0.047
Mild symptom samples	36/36 (100)	31/36 (86)	36/36 (100)	36/36 (100)	36/36 (100)	1.411 ± 0.047
Moderate symptom samples	36/36 (100)	33/36 (92)	36/36 (100)	36/36 (100)	36/36 (100)	1.479 ± 0.038
Severe symptom samples	36/36 (100)	35/36 (97)	36/36 (100)	36/36 (100)	36/36 (100)	1.540 ± 0.052

^a^ Banana pseudostems with mild (necrosis covering less than one-third of the total area of pseudostem), moderate (less than two-thirds but equal to or more than one-third of the total area), and severe (equal to or more than two-thirds of the total area) infection. ^b^ The molecular detection of isolated pathogen (MDIP) assay was performed to confirm infection of 138 field banana pseudostems with *Fusarium oxysporum* (Fo). ^c^ A total of 138 field samples (108 Fo-infected symptomatic samples and 30 Fo-infected but asymptomatic samples) from six different fields were collected for the in planta detection assays.

**Table 4:** Comparison of Fo iiPCR and molecular detection methods for field detection of *Fusarium oxysporum* infection of different age stages of banana plants with mild symptoms. An automated DNA extraction protocol was used.

Age Stage of Banana Plants ^a^	MDIP Assay ^b^	In Planta Detection ^c^				
		cPCR	SYBR-qPCR	Probe-qPCR	iiPCR	
		Detection Rate (%)	Detection Rate (%)	Detection Rate (%)	Detection Rate (%)	S/N Ratio
1	10/10 (100)	9/10 (90)	10/10 (100)	10/10 (100)	10/10 (100)	1.443 ± 0.020
2	10/10 (100)	9/10 (90)	10/10 (100)	10/10 (100)	10/10 (100)	1.411 ± 0.047
3	10/10 (100)	9/10 (90)	10/10 (100)	10/10 (100)	10/10 (100)	1.420 ± 0.051
4	10/10 (100)	9/10 (90)	10/10 (100)	10/10 (100)	10/10 (100)	1.463 ± 0.050

^a^ The age stages of banana plants were 1: seedling (plant height of less than 1.5 m); 2: vegetative (plant height of more than 1.5 m and without flowering); 3: flowering and fruiting; and 4: harvesting (approximately two months after flowering). ^b^ The molecular detection of isolated pathogen (MDIP) assay was performed to confirm infection by *Fusarium oxysporum* (Fo) of 40 field-collected banana pseudostems showing mild symptoms (necrosis covering less than one-third of the total area of pseudostem). ^c^ A total of 40 field samples (10 Fo-infected symptomatic banana plants at each of the four different age stages) from five different fields were collected for the in planta detection assays.

**Table 5:** Reproducibility evaluation of the molecular detection assays used to detect *Fusarium oxysporum* infection of banana plants at different age stages with mild symptoms used as testers.

Age Stage of Banana Plants ^a^	Reproducibility Test (Coefficient of Variation (CV), %) ^b^							
	cPCR		SYBR-qPCR		Probe-qPCR		iiPCR	
	Intraday	Interday	Intraday	Interday	Intraday	Interday	Intraday	Interday
1	46.21 ± 3.24 a	43.8 ± 6.43 a	1.40 ± 0.41 e	1.02 ± 0.40 e	1.83 ± 0.98 e	0.95 ± 0.22 e	3.46 ± 0.69 e	4.72 ± 2.24 de
2	18.10 ± 8.73 bc	20.66 ± 8.58 bc	0.54 ± 0.11 e	0.75 ± 0.14 e	0.47 ± 0.21 e	0.43 ± 0.22 e	4.31 ± 1.39 de	2.99 ± 0.29 e
3	20.25 ± 6.74 bc	24.61 ± 6.16 b	0.64 ± 0.10 e	0.70 ± 0.26 e	0.67 ± 0.09 e	0.55 ± 0.08 e	3.14 ± 2.56 e	2.93 ± 1.74 e
4	16.16 ± 8.66 bcd	11.91 ± 8.19 cde	0.46 ± 0.18 e	0.50 ± 0.12 e	0.60 ± 0.28 e	0.59 ± 0.15 e	2.75 ± 1.62 e	2.45 ± 1.78 e

^a^ The age stages of banana were 1: seedling (plant height of less than 1.5 m); 2: vegetative (plant height of more than 1.5 m and without flowering); 3: flowering and fruiting; and 4: harvesting (approximately two months after flowering). ^b^ Coefficient of variation (CV) is calculated as standard deviation divided by the mean of each assay (n = 40) × 100%. R studio (RStudio, Inc., Vienna, Austria) with the stats package was used to conduct the statistical analyses. All percentage data were subjected to ANOVA, and Tukey’s honestly significant difference (HSD) test was then performed (means indicated by the same letter are not significantly different at the *p* < 0.05 level).

**Table 6:** Comparison of Fo iiPCR and the molecular detection methods for field detection of various *Fusarium oxysporum*-infected asymptomatic and symptomatic banana pseudostem samples using a rapid DNA extraction protocol.

Fo-Infected Samples ^a^	MDIP Assay ^b^	In Planta Detection ^c^				
		cPCR	SYBR-qPCR	Probe-qPCR	iiPCR	
		Detection Rate (%)	Detection Rate (%)	Detection Rate (%)	Detection Rate (%)	S/N Ratio
Symptomless samples	12/12 (100)	7/12 (58)	12/12 (100)	12/12 (100)	12/12 (100)	1.765 ± 0.212
Mild symptom samples	12/12 (100)	9/12 (75)	12/12 (100)	12/12 (100)	12/12 (100)	1.715 ± 0.166
Moderate symptom samples	12/12 (100)	9/12 (75)	12/12 (100)	12/12 (100)	12/12 (100)	1.713 ± 0.178
Severe symptom samples	12/12 (100)	9/12 (75)	12/12 (100)	12/12 (100)	12/12 (100)	1.630 ± 0.208

^a^ Banana pseudostem infection was mild (necrosis covering less than one-third of the total area of a pseudostem), moderate (less than two-thirds but equal to or more than one-third of the total area), or severe (equal to or more than two-thirds of the total area). ^b^ The molecular detection of isolated pathogen (MDIP) assay was performed to confirm infection by *Fusarium oxysporum* (Fo) of 48 field-collected banana pseudostems. ^c^ A total of 48 field samples (36 Fo-infected symptomatic samples and 12 Fo-infected but asymptomatic samples) from six different fields were collected for the in planta detection assays.

**Table 7:** Reproducibility evaluation of molecular detection assays using as testers various *Fusarium oxysporum*-infected asymptomatic and symptomatic banana pseudostem samples extracted by rapid DNA extraction.

Fo-Infected Samples ^a^	Reproducibility Assay (Coefficient of Variation (CV), %) ^b^							
	cPCR		SYBR-qPCR		Probe-qPCR		iiPCR	
	Intraday	Interday	Intraday	Interday	Intraday	Interday	Intraday	Interday
Symptomless samples	53.04 ± 10.86 a	49.40 ± 17.88 a	1.86 ± 0.74 d	1.48 ± 1.08 d	1.09 ± 0.39 d	0.84 ± 0.08 d	6.92 ± 3.51 d	7.35 ± 3.06 d
Mild symptom samples	28.72 ± 29.12 bc	29.12 ± 5.95 bc	0.59 ± 0.50 d	1.06 ± 0.16 d	0.45 ± 0.10 d	0.51 ± 0.08 d	5.57 ± 3.02 d	7.10 ± 2.76 d
Moderate symptom samples	42.08 ± 8.50 ab	27.29 ± 6.86 c	0.49 ± 0.26 d	0.85 ± 0.26 d	0.54 ± 0.15 d	0.40 ± 0.08 d	5.60 ± 1.96 d	6.41 ± 2.26 d
Severe symptom samples	30.07 ± 12.44 bc	28.62 ± 6.00 bc	0.63 ± 0.14 d	0.71 ± 0.24 d	0.42 ± 0.35 d	0.57 ± 0.29 d	7.52 ± 1.18 d	4.80 ± 3.16 d

^a^ Banana pseudostem infection was mild (necrosis covering less than one-third of the total area of pseudostem), moderate (less than two-thirds but equal to or more than one-third of the total area of pseudostem), or severe (equal to or more than two-thirds of the total area of pseudostem). ^b^ Coefficient of variation (CV) is calculated as standard deviation divided by the mean of each assay (n = 48) × 100%. R studio (RStudio, Inc., Vienna, Austria) with the stats package was used to conduct the statistical analyses. All percentage data were subjected to ANOVA, and Tukey’s honestly significant difference (HSD) test was then performed (means indicated with the same letter are not significantly different at the *p* < 0.05 level).

**Table 8:** Comparison of Fo iiPCR and molecular detection methods for field detection of *Fusarium oxysporum* infection of different age stages of banana plants with mild symptoms using a rapid DNA extraction protocol.

Age Stage of Banana Plants ^a^	MDIP Assay ^b^	In Planta Detection ^c^				
		cPCR	SYBR-qPCR	Probe-qPCR	iiPCR	
		Detection Rate (%)	Detection Rate (%)	Detection Rate (%)	Detection Rate (%)	S/N Ratio
1	12/12 (100)	9/12 (75)	12/12 (100)	12/12 (100)	12/12 (100)	1.606 ± 0.174
2	12/12 (100)	9/12 (75)	12/12 (100)	12/12 (100)	12/12 (100)	1.715 ± 0.166
3	12/12 (100)	9/12(75)	12/12 (100)	12/12 (100)	12/12 (100)	1.592 ± 0.171
4	13/13 (100)	9/13 (69)	13/13 (100)	13/13 (100)	13/13 (100)	1.618 ± 0.132

^a^ The age stages of banana plants were 1: seedling (plant height of less than 1.5 m); 2: vegetative (plant height of more than 1.5 m and without flowering); 3: flowering and fruiting; and 4: harvesting (approximately two months after flowering). ^b^ The molecular detection of isolated pathogen (MDIP) assay was performed to confirm infection by *Fusarium oxysporum* (Fo) of the 49 field-collected banana pseudostems showing mild symptoms (necrosis covering less than one-third of the total area of pseudostem). ^c^ A total of 49 Fo-infected symptomatic field samples (12 banana plants at each of the age stages 1–3 and 13 banana plants at age stage 4) from six different fields were collected for the in planta detection assays.

**Table 9:** Reproducibility evaluation of the molecular detection assays using as testers different age stages of banana plants with mild symptoms of *Fusarium oxysporum* infection. The testers were extracted by rapid DNA extraction.

Age Stage of Banana Plants ^a^	Reproducibility Test (Coefficient of Variation (CV), %) ^b^							
	cPCR		SYBR-qPCR		Probe-qPCR		iiPCR	
	Intraday	Interday	Intraday	Interday	Intraday	Interday	Intraday	Interday
1	27.70 ± 8.66 a	27.42 ± 10.77 a	0.51 ± 0.13 d	0.73 ± 0.30 d	0.34 ± 0.04 d	0.44 ± 0.10 d	6.09 ± 2.20 bcd	6.55 ± 3.07 bcd
2	28.72 ± 5.01 a	29.12 ± 5.95 a	0.59 ± 0.50 d	1.06 ± 0.16 d	0.45 ± 0.10 d	0.51 ± 0.08 d	5.57 ± 3.02 cd	7.10 ± 2.76 bcd
3	19.53 ± 11.37 abc	33.48 ± 12.11 a	0.62 ± 0.21 d	0.46 ± 0.14 d	0.35 ± 0.10 d	0.24 ± 0.10 d	4.85 ± 1.99 cd	5.37 ± 0.65 cd
4	23.05 ± 3.81 a	21.09 ± 10.81 ab	0.82 ± 0.28 d	0.34 ± 0.22 d	0.46 ± 0.05 d	0.63 ± 0.30 d	5.27 ± 2.27 cd	5.75 ± 1.17 cd

^a^ The age stages of banana were 1: seedling (plant height of less than 1.5 m); 2: vegetative (plant height of more than 1.5 m and without flowering); 3: flowering and fruiting; and 4: harvesting (approximately two months after flowering). ^b^ Coefficient of variation (CV) is calculated as standard deviation divided by the mean of each assay (n = 49) × 100%. R studio (RStudio, Inc., Vienna, Austria) with the stats package was used to conduct the statistical analyses. All percentage data were subjected to ANOVA, and Tukey’s honestly significant difference (HSD) test was then performed (means indicated by the same letter are not significantly different at the *p* < 0.05 level).

**Table 10:** Comparison of the molecular detection protocols using asymptomatic and symptomatic *Fusarium oxysporum*-infected samples extracted by automated DNA extraction or rapid DNA extraction as testers.

Protocols/Samples Used	Precision ^a^	Recall ^b^	Accuracy ^c^	F1 Score ^d^
Asymptomatic samples				
Automatic DNA extraction protocol–cPCR	1.000	0.667	0.667	0.800
Automatic DNA extraction protocol–SYBR-qPCR	1.000	1.000	1.000	1.000
Automatic DNA extraction protocol–Probe-qPCR	1.000	1.000	1.000	1.000
Automatic DNA extraction protocol–iiPCR	1.000	1.000	1.000	1.000
Rapid DNA extraction protocol–cPCR	1.000	0.583	0.583	0.737
Rapid DNA extraction protocol–SYBR-qPCR	1.000	1.000	1.000	1.000
Rapid DNA extraction protocol–Probe-qPCR	1.000	1.000	1.000	1.000
Rapid DNA extraction protocol–iiPCR	1.000	1.000	1.000	1.000
Symptomatic samples				
Automatic DNA extraction protocol–cPCR	1.000	0.913	0.913	0.955
Automatic DNA extraction protocol–SYBR-qPCR	1.000	1.000	1.000	1.000
Automatic DNA extraction protocol–Probe-qPCR	1.000	1.000	1.000	1.000
Automatic DNA extraction protocol–iiPCR	1.000	1.000	1.000	1.000
Rapid DNA extraction protocol–cPCR	1.000	0.740	0.740	0.850
Rapid DNA extraction protocol–SYBR-qPCR	1.000	1.000	1.000	1.000
Rapid DNA extraction protocol–Probe-qPCR	1.000	1.000	1.000	1.000
Rapid DNA extraction protocol–iiPCR	1.000	1.000	1.000	1.000

^a^ Precision = True positives/(True positives + False positives). ^b^ Recall = True positives/(True positives + False negatives). ^c^ Accuracy = (True positives + True negatives)/(True positives + False positives + False negatives + True negatives). ^d^ F1 score is the harmonic mean of precision and recall; F1 score = 2 × Precision × Recall/(Precision + Recall).

**Table 11:** Comparison of Fo iiPCR-, Fo qPCR-, and Fo cPCR-based in planta detection for field diagnosis.

Associated Pathogen	Name of Marker ^a^	Amplification Primers	
		Names	Sequences (5′-3′)
*Fusarium oxysporum*	iiFoc_104_	LNHFnF-1 LNHFnR-1	CAGGGGATGTATGAGGAGGCTA CGGAAACAGACTCTTGCCATTC
All fungal pathogens	ITS1-5.8S-ITS2	ITS1 ITS4	TCCGTAGGTGAACCTGCGG TCCTCCGCTTATTGATATGC
*F. oxysporum*	Fn_327_	FnSc-1 FnSc-2	TACCACTTGTTGCCTCGGCGGATCAG TTGAGGAACGCGAATTAACGCGAGTC

^a^ All markers target the same locus in this study.

## Data Availability

The data presented in this study are openly available online.
